# Recurrent Prostate Cancer Diagnostics with ^18^F-PSMA-1007 PET/CT: A Systematic Review of the Current State

**DOI:** 10.3390/diagnostics12123176

**Published:** 2022-12-15

**Authors:** Laura Saule, Maija Radzina, Mara Liepa, Lilita Roznere, Andrejs Lioznovs, Madara Ratniece, Edgars Mamis, Egils Vjaters

**Affiliations:** 1 Radiology Research Laboratory, Riga Stradins University, LV-1007 Riga, Latvia; 2 Diagnostic Radiology Institute, Paula Stradina Clinical University Hospital, LV-1002 Riga, Latvia; 3 Medical Faculty, University of Latvia, LV-1004 Riga, Latvia; 4 Center of Urology, Paula Stradina Clinical University Hospital, LV-1002 Riga, Latvia

**Keywords:** F-PSMA, PET/CT, prostate cancer, local recurrence, lymph nodes, bone metastases, biochemical relapse

## Abstract

Background: Early diagnosis of recurrent prostate cancer is a cornerstone for further adequate therapy planning. Therefore, clinical practice and research still focuses on diagnostic tools that can detect prostate cancer in early recurrence when it is undetectable in conventional diagnostic imaging. ^18^F-PSMA-1007 PET/CT is a novel method to evaluate patients with biochemical recurrent PCa. The aim of this review was to evaluate the role of ^18^F-PSMA-1007 PET/CT in prostate cancer local recurrence, lymph node metastases and bone metastases detection. Methods: Original studies, reviews and five meta-analyses were included in this article. A total of 70 studies were retrieved, 31 were included in the study. Results: All patients described in the studies underwent ^18^F-PSMA-1007 PET/CT. The administered ^18^F-PSMA-1007 individual dose ranged from 159 ± 31 MBq to 363.93 ± 69.40 MBq. Results showed that ^18^F-PSMA-1007 PET/CT demonstrates a good detection rate in recurrent prostate cancer. Conclusions: ^18^F-PSMA-1007 PET/CT appears to achieve reliable performance in detecting recurrent prostate cancer. The high detection rate of ^18^F-PSMA-1007 PET/CT in recurrent prostate cancer was confirmed, especially in local recurrence and small lymph nodes with non-specific characteristics on conventional diagnostic imaging methods. However, several authors emphasize some limitations for this tracer—for example, non-specific uptake in bone lesions that can mimic bone metastases.

## 1. Introduction

Prostate cancer (PCa) is one of the most common malignancies that affect men worldwide. Prostate cancer is the second most commonly occurring cancer in men and the fourth most common cancer overall [1]. A total of 1,414,259 new cases of prostate cancer and 375,304 related deaths were reported in 2020 globally [2]. A systematic review of autopsy studies reported a prevalence of PCa at age <30 years of 5% (95% confidence interval [CI]: 3–8%), increasing by an odds ratio (OR) of 1.7 (1.6–1.8) per decade, to a prevalence of 59% (48–71%) by age >79 years [3]. The main radical treatment methods for primary prostate cancer are prostatectomy and radiation therapy. Despite that, recurrence of the PCa is frequent and patients with clinically intermediate or high-risk prostate cancer after initial radical treatment need clinical follow-up. Biochemical recurrence is when PSA levels rise in the blood to a certain threshold after prostate cancer treatment—radical prostatectomy or radiation therapy [4]. The biochemical persistence is defined as a PSA level persistence/recurrence after radical prostatectomy such that the PSA fails to fall to undetectable levels [5].

Serum prostate-specific antigen (PSA) is a biomarker for prostate cancer screening and a reliable marker of PCa recurrence after initial treatment.

Prostate specific membrane antigen (PSMA) is a glycoprotein that is hyper expressed in prostate cancer tissues while its degree of expression correlates with tumor aggressiveness, metastatic disease and disease recurrence. In recent years, PSMA positron emission tomography/computed tomography (PET/CT) has become the gold standard for the staging of primary prostate cancer and restaging biochemical recurrent prostate cancer patients. PSMA labeled radioligands have outperformed conventional imaging modalities, such as computed tomography (CT), magnetic resonance imaging (MRI) and bone scintigraphy, and previous generation radiopharmaceuticals [6,7,8].

PET/CT is a functional and anatomical non-invasive radiological hybrid imaging modality. Several radiopharmaceuticals with different diagnostic sensitivity and specificity are used in the detection and staging of prostate cancer, in the evaluation of treatment efficacy and localization of recurrence [9].

^18^F-PSMA-1007 is a novel PSMA-based radiopharmaceutical. Fluorine-18 (^18^F) labelled PSMA radiotracers have several advantages over Gallium-68 (^68^Ga) labelled radioligands: ^18^F is a cyclotron produced isotope with a longer half live and a lower positron energy compared to ^68^Ga, which leads to improved spatial resolution [10]. Additionally, due to non-urinary excretion, studies with ^18^F-PSMA-1007 have improved detection rates especially in local relapses and pelvic lymph node metastases in proximity to the urinary tract [8,11].

According to several publications, ^18^F-PSMA-1007 PET/CT tests are highly valuable for detecting prostate cancer biochemical recurrences. Giesel et al. analyzed 251 patients, and 204 (81.3%) of them had evidence of recurrence on ^18^F-PSMA-1007 PET/CT examination. The detection rates (DR) were 94.0% (for PSA levels greater than or equal to 2), 90.9% (for PSA levels 1 to less than 2), 74.5% (for PSA levels 0.5 to less than 1), and 61.5% (for PSA levels 0.2 to less than 0.5 ng/mL) [12]. Sprute et al. conducted a study using ^18^F-PSMA-1007 PET/CT on 96 patients with prostate cancer. In this study ^18^F-PSMA-1007 PET/CT had a lesion-based sensitivity of 81.7%, a specificity of 99.6%, a positive predictive value (PPV) of 92.4%, and a negative predictive value (NPV) of 98.9% for detecting positive lymph nodes larger than 3 mm [13]. When compared to bone scintigraphy (BS), ^18^F-PSMA-1007 PET/CT was more effective in detecting small lesions with minimal osteosclerosis or excluded degenerative change although bone scintigraphy is sufficiently sensitive to detect small bone metastasis in most of the recurrent cases [14].

Despite the specificity of ^18^F-PSMA-1007, physiological uptake of this radiotracer can be seen in the salivary glands, gallbladder, prostate, kidneys, liver, lacrimal glands, spleen and small intestine [15]. However, concentrated foci with localized abnormal radioactivity uptake are considered positive, such as avid uptake in lymph nodes and bones, which can be diagnosed as metastases [16].

The aim of this review was to evaluate the role of ^18^F-PSMA-1007 PET/CT in patients with biochemically recurrent prostate cancer local recurrence, lymph node and bone metastases detection.

## 2. Materials and Methods

In the present paper, a comprehensive literature search of PubMed, Google Scholar and Scopus databases was conducted with key words: “recurrent” “prostate” “cancer” “F-PSMA”. The results were subdivided in subjects and classified in “prostate cancer local recurrence”, “prostate cancer lymph node metastases” and “prostate cancer bone metastases” subdivisions. Additionally, articles about ^18^F-PSMA-1007 characteristics and dose protocol were studied. The search was updated from 2018 until September of 2022 and references of the retrieved articles were explored. Original studies, reviews and five meta-analyses were included in this article. A total of 70 studies were retrieved, 31 were included in the study. The inclusion criteria for the relevant studies were as follows: (1) all patients underwent ^18^F-PSMA-1007 PET/CT in the study; (2) subjects were also diagnosed with PCa by histopathology, other imaging examinations or clinical follow-up; (3) analyses were performed on a per-patient or per-lesion basis.

## 3. Results

### 3.1. ^18^F-PSMA-1007 Radiotracer Characteristics

PSMA is a type II transmembrane glycoprotein with enzymatic carboxypeptidase activity that is expressed in the cytosol of normal prostatic cells. PSMA is highly overexpressed on the membrane of prostate cancer cells. The intensity of membranous PSMA expression correlates positively with tumor grade, rises under androgen deprivation and in metastatic and castration-resistant cancer, thus rendering it an appropriate target for imaging and treatment [17].

PSMA is an excellent target for several reasons: preferential, marked overexpression by most PCa cells, positive correlation of its expression with tumor grade and disease stage, low presence in the bloodstream by virtue of its transmembrane localization, and internalization and retention within tumor cells after binding to its ligand. Moreover, in personalized medicine there is increased interest in the use of PSMA for therapeutic approaches—in the theranostics setting, that means combining imaging diagnosis with targeted radionuclide therapy [17,18].

Several PSMA ligands, differing slightly in chemical structure, are commercially available and they may be radiolabeled with different positron-emitting isotopes as Gallium-68 (^68^Ga), Fluorine-18 (^18^F) or Copper-64 (^64^Cu) to obtain PET radiopharmaceuticals used in clinical practice. Labeling of PSMA agents with ^18^F may offer numerous advantages, including longer half-life and improved image resolution. Due to the lower positron energy, the theoretical achievable resolution of ^18^F is slightly better in comparison to ^68^Ga [19,20,21,22,23,24]. Studies with ^18^F-PSMA-1007 also suggest improved detection rates especially in local relapses and pelvic lymph node metastases in proximity to the urinary tract when compared to ^68^Ga-PSMA-11 PET/CT [11].

The ^18^F-PSMA-1007 seems to be more favorable among other ^18^F-PSMA ligands candidate compounds because it demonstrates high labeling yields, better tumor uptake and non-urinary background clearance [25]. In ^18^F-PSMA-1007 PET/CT, physiological uptake can be seen in the liver, gallbladder, prostate, kidneys, salivary glands, lacrimal glands, spleen and small intestine [15].

In several studies no drug-related pharmacological effects or physiologic responses were not reported in the patients. All observed parameters (e.g., blood pressure, heart rate and body temperature) remained normal and unchanged during and after the ^18^F-PSMA-1007 PET/CT examination. Overall, no adverse events due to ^18^F-PSMA-1007 administration were reported in the included studies. No patient reported subjective symptoms [15,26].

Chemical structures of different PSMA ligands are compared in the Table 1 [11,17,27].

### 3.2. ^18^F-PSMA-1007 PET/CT Scanning Protocol

All patients described in the studies underwent ^18^F-PSMA-1007 PET/CT. The time interval between injection and image acquisition varies from 57.7  ±  4.9 min to 120 ± 10 min after injection of ^18^F-PSMA-1007. The ^18^F-PSMA-1007 solution was given by intravenous bolus injection [8,14].

All patients received regular whole-body ^18^F-PSMA-1007 PET/CT scans (in the most cases from head to the thighs). The popular scanner model was Biograph-mCT PET/CT (Siemens, Erlangen, Germany) [8,12,15,25,28]. The administered ^18^F-PSMA-1007 individual dose ranged from 159 ± 31 MBq to 363.93 ± 69.40 MBq [28,29]. It was confirmed that ^18^F-PSMA-1007 can be safely administered and results in a mean effective dose of 12.8 ± 0.6 μSv/MBq. Therefore, the total radiation dose is lower than for other PSMA PET agents and in the same range as ^18^F-DCFPyL [30].

In most of the studies all images were interpreted by two physicians—radiologists and/or nuclear medicine physicians in consensus. Image analysis was performed using an appropriate workstation and software. Focal uptake of ^18^F-PSMA-1007 higher than the surrounding background and not associated with physiologic uptake was considered suggestive of malignancy. Moreover, typical pitfalls in PSMA ligand PET imaging (e.g., uptake in celiac and other ganglia, fractures and degenerative changes) were considered. If at least one PSMA positive lesion suspicious for PCa was described, the PET/CT was counted as positive [8,12,31].

All technical aspects of ^18^F-PSMA-1007 PET/CT in the included studies are described in Table 2. The included studies in the Table 2 were selected based on following criteria: PET/CT examination with ^18^F-PSMA-1007 radiotracer; patient cohort was recurrent prostate patients; and technical aspects were fully reflected in the article.

### 3.3. Local Recurrence

**Figure 1:**
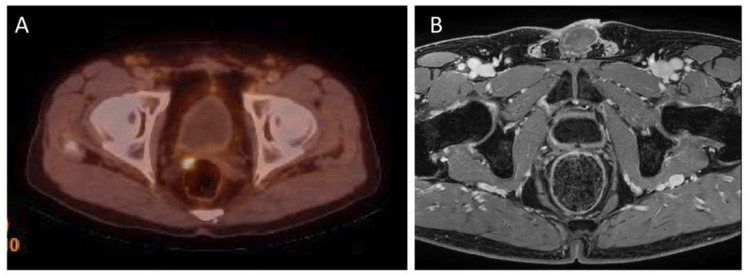
(**A**)—^18^F-PSMA-1007 PET/CT examination of a patient (74 years old) after radical prostatectomy eight years before, Gleason Score 7(3 + 4), current PSA level 0.29 ng/mL. ^18^F-PSMA-1007 focal uptake in right side tissues adjacent to the prostate bed, in the level under seminal vesicle, pararectaly with SUVmax = 6.9 was detected, confirming local recurrence. (**B**) this lesion cannot be seen on the corresponding MRI examination (Images from Riga Stradins University Radiology research laboratory archive).

Local recurrence of prostate cancer is defined as focal uptake of ^18^F-PSMA-1007 radiotracer higher than the surrounding background and not associated with physiologic uptake in PET/CT. It has been reported that the detection rate (DR) of ^18^F-PSMA-1007 PET/CT in local recurrence is related to PSA serum values. Results of the meta-analysis by Ferrari et al. confirmed that ^18^F-PSMA-1007 PET/CT demonstrated a good detection rate in biochemical recurrent prostate cancer (81.3%) and higher PSA values were associated with higher DR [38]. In addition, in a study by Lengana et al., the detection rate depending on the PSA level was determined. The detection rates for PSA levels 0–<0.5 ng/mL were 31.3% for 0.5–<1 ng/mL—33.3%, for 1–2 ng/mL detection rate were 55.6% and for PSA level > 2 ng/mL detection rate were 72.2%. In this study 7 (29.2%) of the positive patients had been described as negative or equivocal on conventional imaging. In addition, an optimal PSA cut-off level was determined—1.3 ng/mL [39]. See Figure 1 for an example of local recurrence in the prostate bed in PET/CT.

Giesel et al. conducted a retrospective multicenter study with 251 patients, with a median age of 70 years. Of these 251 patients, 204 (81.3%) had evidence of recurrence on ^18^F-PSMA-1007 PET/CT. The detection rates were 94.0% (PSA levels of greater than or equal to 2 ng/mL), 90.9% (PSA level 1 ng/mL to less than 2 ng/mL), 74.5% (PSA level 0.5 ng/mL to less than 1 ng/mL) and 61.5% (PSA level 0.2 ng/mL to less than 0.5 ng/mL). The overall detection rate was 80.2% [12]. Similar results were obtained in a meta-analysis by Treglia et al.—the pooled DR of ^18^F-PSMA-1007 PET/CT in biochemical prostate cancer recurrence patients was 81% (95% CI: 71–88%). The pooled DR was 86% for PSA level ≥ 0.5 ng/mL (95% CI: 78–93%) and 49% for PSA level < 0.5 ng/mL (95% CI: 23–74%). It was concluded that detection rate of ^18^F-PSMA-1007 PET/CT is related to PSA values with significant lower DR in patients with PSA < 0.5 ng/mL [23].

In a study by Watabe et al., 28 patients with biochemical recurrent prostate cancer underwent ^18^F-PSMA-1007 PET/CT. Biochemical recurrence was defined as a continuous increase in PSA after radical prostatectomy or radiation therapy without any apparent recurrent lesions on conventional diagnostic imaging. The detection rates were 66.7% (PSA level 0.1–0.5 ng/mL), 85.7% (PSA level 0.5–1.0 ng/mL) and 100% (PSA level above 1.0 ng/mL). In 53% of biochemical recurrent prostate cancer patients who were suspected of local recurrence, focal uptake was detected adjacent to the bladder on ^18^F-PSMA-1007 PET/CT. This suggested at significant advantage of ^18^F-PSMA-1007 as compared to other PSMA ligands due to minimal physiological urine excretion [14].

The maximum standardized uptake value (SUVmax) is widely used for measuring the uptake of tracer by malignant tissue [40]. Increased ^18^F-PSMA-1007 uptake values reflect the viability of cancer cells, and can be imaged and quantified using PET/CT. It was proved that mean prostate maximum standardized uptake value (SUVmax) was significantly higher in prostate cancer than in benign lesions (19.56 ± 18.11 vs. 4.21 ± 1.5, *p* = 0.00001), in patients with PSA > 20 ng/mL versus PSA < 20 ng/mL (19.1 ± 20.6 vs. 6.01 ± 5.4, *p*—0.0052) and in patients with Gleason’s score (GS) score > 7 vs. GS ≤ 7 (28.1 ± 20.3 vs. 10.2 ± 8.9, *p*—0.010). In that study, Chandra et al. concluded that PSMA PET/CT can differentiate benign and malignant lesions of the prostate with very high accuracy [41].

In several studies, the detection rate of ^18^F-PSMA-1007 PET/CT was compared with ^68^Ga-PSMA-11 PET/CT. Lengana et al. conducted a prospective study where ^18^F-PSMA-1007 PET/CT was performed in the same patients after ^68^Ga-PSMA-11 PET/CT examination. Local recurrence diagnosed on each of these studies was compared against the final diagnosis based on a clinical follow-up and histological correlation. ^18^F-PSMA-1007 PET/CT was able to detect more sites of recurrence as compared to ^68^Ga-PSMA-11 PET/CT; the sites were mainly localized within the prostate and surrounding pelvic structures [42]. In a study conducted by Hoffmann et al., ^18^F-PSMA-1007 PET/CT showed prostate cancer lesions in 87.5%, while ^68^Ga-PSMA-11 PET/CT identified them in 88.9%. In this study for ^18^F-PSMA-1007 PET/CT biochemical recurrent patients who were treated with radical prostatectomy, a PSA level of 1.08 ng/mL was found to be the optimal cut-off level for predicting positive and negative scans [32].

In a meta-analysis conducted by Liu et al., the pooled sensitivity and specificity of ^18^F-PSMA-1007 PET/CT in prostate cancer were 0.836 and 0.946, respectively. The final analysis included 11 studies that described 799 patients and 4261 lesions in ^18^F-PSMA-1007 PET/CT. This meta-analysis showed that ^18^F-PSMA-1007 PET/CT has a higher diagnostic efficacy for prostate cancer both in the setting of primary prostate cancer and biochemical recurrence, as compared to other PSMA ligands. It was concluded—when serum PSA level increases, the diagnostic accuracy of ^18^F-PSMA-1007 PET/CT also improves [43].

De Man et al. conducted a prospective observational study in which 60 prostate cancer patients (9 primary staging, 51 biochemical recurrence) were imaged with ^18^F-PSMA-1007 PET/CT. In this study, pre-scan and post-scan questionnaires were completed by the treating physician to observe changes in therapy intent. The patient-based detection rate was 82% and a management change was seen in 52% of the cases. The heterogeneous characteristics of the included patients resulted in a widely varying treatment change, mostly originating from an increase in disease extent on ^18^F-PSMA-1007 PET/CT [44].

Another meta-analysis by Wang et al. investigated the diagnostic performance of PET/CT using three different ^18^F-labeled radiotracers—^18^F-labeled choline, fluciclovine and PSMA—in patients with biochemical recurrent prostate cancer. It was concluded that ^18^F-PSMA PET/CT demonstrated a significantly higher detection rate over ^18^F-labeled choline and ^18^F-labeled fluciclovine for different PSA levels, particularly in PSA levels less than 2.0 ng/mL [45].

Another meta-analysis by Liu et al. concluded that ^18^F-PSMA-1007 PET/CT had a high application value for prostate cancer, including primary tumors and biochemical recurrence. The final analysis included 15 studies that described 1022 patients and 2034 lesions with ^18^F-PSMA-1007 PET/CT in prostate cancer. The DR of ^18^F-PSMA-1007 PET/CT in patients with biochemical recurrent prostate cancer ranged from 47% to 100%, with a pooled estimated DR of 86% [46].

In a retrospective study by Mingels et al., which included 177 consecutive patients who underwent ^18^F-PSMA-1007 PET/CT, a total of six body regions were defined: prostate fossa, pelvic lymph nodes (LN), retroperitoneal LN, supradiaphragmatic LN, bones and soft tissue. Sensitivity and specificity for local recurrence were 0.94 (CI: 0.81–0.98) and 0.92 (CI: 0.64–0.99), respectively. In this study, the known high PET positivity rate of ^18^F-PSMA-1007 PET/CT in recurrent prostate cancer was confirmed [8].

It has been reported that the detection rate of ^18^F-PSMA-1007 PET/CT is higher than CT alone in the evaluation of biochemical recurrence of prostate cancer after radical prostatectomy. A study conducted by Morawitz et al. included 59 prostate cancer patients. ^18^F-PSMA-1007 PET/CT and CT images were evaluated separately regarding prostate cancer lesion count, type and localization by two physicians. ^18^F-PSMA-1007 PET/CT detected 141 lesions (99.3%) in 50 patients (84.7%), while CT detected 72 lesions (50.7%) in 29 patients (49.2%). A significantly higher detection rate of ^18^F-PSMA-1007 PET/CT was observed on a lesion-based analysis (*p* < 0.0001) and on a patient-based analysis (*p* < 0.0001). It has been confirmed that ^18^F-PSMA-1007 PET/CT is superior to CT alone in detecting biochemical recurrence in prostate cancer patients after radical prostatectomy and offers additional therapeutic options in a relevant number of patients [24].

Rauscher et al. conducted a retrospective study where the frequency of non-tumor-related uptake and the detection efficacy in recurrent prostate cancer patients in ^18^F-PSMA-1007 PET/CT and ^68^Ga-PSMA-11 PET/CT were evaluated. ^18^F-PSMA-1007 PET/CT revealed approximately five times more lesions were attributed to a benign origin than ^68^Ga-PSMA-11 PET/CT revealed (245 vs. 52 lesions, respectively). The most frequently observed benign lesions were ganglia, nonspecific lymph nodes and bone lesions. The number of lesions with increased PSMA ligand uptake attributed to a benign origin is considerably higher for ^18^F-PSMA-1007 PET/CT than for ^68^Ga-PSMA-11 PET/CT. It was concluded that this finding indicates the need for sophisticated reader training emphasizing known pitfalls and reporting within the clinical context [28].

A comparison of sensitivity, specificity and detection rate of local recurrence of prostate cancer is shown in Table 3.

### 3.4. Lymph Node Metastases

**Figure 2:**
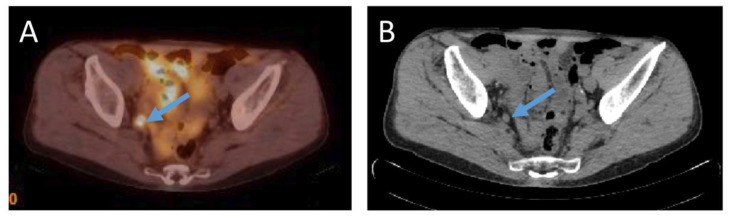
(**A**)—^18^F-PSMA-1007 PET/CT examination of a patient (74 years old) after radical prostatectomy ten years before, Gleason Score 6 (3 + 3), current PSA level 4.77 ng/mL. ^18^F-PSMA-1007 uptake in an 8 mm in size obturatory right side lymph node with SUVmax = 13.7 was detected. (**B**) an 8 mm in size round shaped obturatory right side lymph node in corresponding computed tomography image in axial plane (Images from Riga Stradins University Radiology research laboratory archive).

Lymph nodes adjacent to the primary tumor are often the first site of metastases. Additionally, lymph node metastases are commonly detected in prostate cancer recurrence. Detection of lymph node metastasis is one of major prognostic significance for prostate cancer, although lymph node metastases are themselves rarely life threatening [47,48]. See Figure 2 for example of lymph node metastasis.

Sprute et al. conducted a study using ^18^F-PSMA-1007 PET/CT on 96 patients with prostate cancer—90.6% received ^18^F-PSMA-1007 PET/CT for staging before the primary treatment, whereas 9.4% underwent imaging for biochemical recurrence. ^18^F-PSMA-1007 PET/CT had sensitivity of 81.7%, a specificity of 99.6%, PPV of 92.4% and NPV of 98.9% for detecting positive lymph nodes larger than 3 mm [13]. A similar study was conducted by Giesel et al. in which 251 patients were analyzed. Lymph node metastases were present in the pelvis in 40.6% of patients (*n* = 102), in the retroperitoneum in 19.5% of patients (*n* = 49), but in supradiaphragmatic locations in 12.0% of patients (*n* = 30) [12].

The role of ^18^F-PSMA-1007 PET/CT in detection of lymph node metastases is to detect small metastases which were undetectable by conventional imaging methods, such as computed tomography. In a study conducted by Watabe et al., a total of 28 patients with biochemical prostate cancer recurrence were enrolled. Biochemical recurrence was defined as a continuous increase in PSA after radical prostatectomy or radiation therapy without any apparent recurrent lesions on conventional diagnostic imaging (CT and bone scintigraphy). In biochemical recurrent prostate cancer patients who had negative findings on conventional CT or bone scintigraphy, the PSMA positive lesions were mainly smaller lymph nodes or nodules undetectable by CT [14].

A similar case study was conducted by Mori et al. in which the case of an 83-year-old man with castration resistant prostate cancer was reported. Strong accumulation of ^18^F-PSMA-1007 in PET/CT was seen not only in the left internal iliac lymph node but also in the two obturatory lymph nodes that could not be detected with conventional CT or magnetic resonance imaging. After the diagnosis of oligometastases in the pelvic lymph nodes, the patient underwent laparoscopic pelvic lymph node dissection, which revealed lymph node metastases in two obturatory lymph nodes (in the left side) and the left internal iliac lymph node, corresponding to the ^18^F-PSMA-1007 accumulation sites [49].

In a study by Saule et al., 28 patients with biochemical prostate cancer recurrence underwent ^18^F-PSMA-1007 PET/CT in addition to a pelvic multiparametric MRI and bone scintigraphy within ≤3 months before or ≤1 month after PET/CT were performed as components of their clinical routine work-up. Against the standard of reference, sensitivity, specificity, accuracy, positive predictive value and negative predictive value of PET/CT for local lymph node metastases were 92.3%, 93.3%, 92.9%, 92.3% and 93.3%, respectively. In MRI, sensitivity, specificity, accuracy, PPV and NPV for lymph node metastases were 53.8%, 100%, 78.6%, 100% and 71.4%, respectively. It was concluded that the detection of regional lymph node metastases ^18^F-PSMA-1007 PET/CT provides superior sensitivity and overall diagnostic accuracy compared to MRI, in particular for small lymph nodes with non-specific characteristics on MRI [29].

In addition, the result of the preoperative ^18^F-PSMA-1007 PET/CT can be a predictor of biochemical persistence and early recurrence after radical prostatectomy with extended pelvic lymph node dissection (ePLND). Baas et al. conducted a study with the aim to evaluate the predictive value of lymph nodes suspicious for metastases on preoperative ^18^F-PSMA-1007 PET/CT for biochemical persistence and early biochemical recurrence following robotic-assisted radical prostatectomy with ePLND. Patients were grouped as PSMA positive or PSMA negative depending on their lymph node status on ^18^F-PSMA-1007 PET/CT and subdivided according to histological lymph node status in pN0 or pN1. Results of this study show that the presence of PSMA positiveness was a significant predictor for biochemical persistence (OR 7.1, 2.9–17.1 95% CI) and for biochemical recurrence (OR 8.1, 2.9–22.6 95% CI). It was concluded that preoperative PSMA-PET/CT may be a valuable tool for patient counseling for robotic-assisted radical prostatectomy and extended pelvic lymph node dissection as it is a significant predictor for the risk of postoperative biochemical persistence and early biochemical recurrence. It was also concluded that an ePLND should not be avoided in men with intermediate or high-risk prostate cancer and preoperative negative ^18^F-PSMA-1007 PET/CT, as 20% have microscopic lymph node metastasis [50].

### 3.5. Bone Metastases

**Figure 3:**
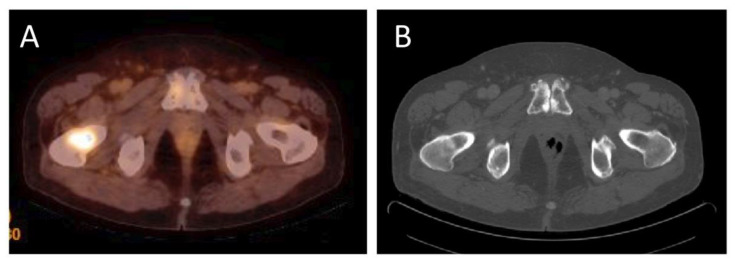
(**A**)—^18^F-PSMA-1007 PET/CT examination of a patient (69 years old) after radical prostatectomy seven years before, current PSA level 3.9 ng/mL. ^18^F-PSMA-1007 uptake in the right femur with SUVmax = 5.7 was detected to approve metastatic activity. (**B**) in computed tomography mild, local sclerotic lesion in the right femoral neck was inconclusive (Images from Riga Stradins University Radiology research laboratory archive).

Bone metastases are typical in advanced stages of prostate cancer. Imaging of bone metastases is important for localization and characterization, and for evaluation of their size and number and for follow-up after therapy. Bone metastases formation is triggered by cancer initiating cells in the bone marrow and is facilitated by the release of several growth factors [19]. Figure 3 shows an example of bone metastasis.

Watabe et al. conducted a study in which biochemical prostate cancer recurrence was defined as a continuous increase in PSA level after radical prostatectomy or radiation therapy without any apparent recurrent lesions on conventional diagnostic imaging (CT and bone scintigraphy). In this study, results show that the SUVmax was 4.1  ±  1.6 in bone metastasis. Among the PET positive biochemical prostate cancer recurrence patients (*n*  =  26), bone metastases were detected in 15.4% (4/26). When compared to bone scintigraphy, ^18^F-PSMA-1007 PET/CT was more effective in detecting small lesions with minimal osteosclerosis or excluding degenerative changes, although bone scintigraphy (BS) is sufficiently sensitive to detect small bone metastasis in most of the recurrent cases. It is reported that caution is advised when interpreting bone uptake of ^18^F-PSMA-1007, especially in the ribs, due to potential false-positive findings [14,27].

In a study conducted by Ahmadi Bidakhvidi et al., several parameters predicting ^18^F-PSMA-1007 PET/CT scan positivity in biochemical recurrent prostate cancer patients were analyzed. As a result, PSA velocity and PSA value were significantly correlated with the number of involved bone, lymph node and soft tissue lesions in univariable analysis, but multivariable analysis confirmed PSA value as an independent predictor of the number of bone lesions (IRR 1.003, *p*  =  0.0002). It was concluded that a higher pathological primary tumor staging was significantly correlated with a lower number of bone lesions in multivariable analysis [33].

In another study conducted by Wondergem et al., 120 consecutive patients scanned with ^18^F-PSMA-1007 PET/CT were match-paired with 120 patients scanned with ^18^F-DCFPyL PET/CT. In this study all 240 PET/CT scans were reviewed by two readers and scored according to the criteria of the PSMA Reporting and Data System. Inter-reader agreement and the detection rate for suspected lesions were scored for different anatomic locations, such as the prostate, prostatic fossa, lymph nodes and bone. Regarding suspected bone lesions, almost perfect inter-reader agreement was found at all localizations for ^18^F-DCFPyL. For ^18^F-PSMA-1007, lower inter-reader agreement was found for lesions in the thoracic region and whole skeleton and to a lesser extent for suspected bone lesions in the pelvis. Both readers scored a significantly greater number of equivocal bone lesions in the thoracic region with ^18^F-PSMA-1007 at the expense of the number of scans without bone lesions [37].

In a study conducted by Dietlein et al., intraindividual comparison of ^18^F-PSMA-1007 PET/CT with other PSMA ligands (renally excreted) in patients with recurrent prostate cancer was performed. In this study participated 27 patients whose PET/CT results were obtained with ^68^Ga-PSMA-11, ^18^F-DCFPyL or ^18^F-JK-PSMA-7 and were interpreted as equivocal or negative or as oligometastatic disease. Within 3 weeks, a second PET scan with ^18^F-PSMA-1007 was performed. The confidence in the interpretation of PSMA positive regional findings was scored on a 5 point scale, first in routine diagnostics and then by an independent second evaluation. Discordant PSMA positive skeletal findings were examined by contrast-enhanced MRI. It was explored that ^18^F-PSMA-1007 PET/CT detected a significantly higher number of PSMA-positive bone marrow findings than did PET/CT with 3 other tracers. However, ^18^F-PSMA-1007 exhibits unspecific PSMA tracer accumulation in the bone marrow in a relevant number of patients. Therefore, skeletal lesions, which were detected with ^18^F-PSMA-1007 PET/CT, require verification through MRI or simultaneous PET/MRI. Imaging with ^18^F-PSMA-1007 PET/CT may therefore be applicable primarily to patients with a high probability of locally restricted disease or as a follow-up test in cases with equivocal findings adjacent to the urinary tract. When there is suspicion on distant metastases, particularly in the bone marrow, ^68^Ga-PSMA-11, ^18^F-DCFPyL or ^18^F-JK-PSMA-7 may be more suitable because of their higher specificity in the bone marrow [27].

Another author emphasizes the same limitations for this tracer—nonspecific uptake in bone lesions. In a study by Mingels et al., a lower positive predictive value (PPV) for bone lesions in ^18^F-PSMA-1007 PET/CT was observed, in line with previous reports of high rates of non-specific bone uptake. Sensitivity and specificity for bone lesions were 0.97 (CI: 0.85–0.99) and 0.74 (CI: 0.57–0.85) in this study [8].

One more example that can mimic bone metastases is rib fractures. In a case study by Panagiotidis et al. rib fractures were mimicking bone metastases in ^18^F-PSMA-1007 PET/CT. In this case of a 69-year-old man with a history of prostate adenocarcinoma and suspected biochemical recurrence, PET/CT showed ^18^F-PSMA-1007 uptake in healing rib fractures with no other pathologic findings. Clinicians reporting ^18^F-PSMA-1007 PET/CT should be aware of this potential pitfall, especially in nontypical trauma pattern (for example, solitary osseous lesion) mimicking bone metastasis [51].

## 4. Discussion

Recently, ^18^F-PSMA-1007 PET/CT has become an indispensable part in the management of recurrent prostate cancer patients overcoming the challenges of low sensitivity and specificity of conventional imaging modalities. This review mainly focused on evaluating the diagnostic value of ^18^F-PSMA-1007 PET/CT in prostate cancer patients with biochemical recurrence in evaluation of local recurrence, lymph node metastases and bone metastases.

^18^F-PSMA-1007 is one of the most promising radiotracers for PET/CT imaging of recurrent prostate cancer. The current guidelines of the European Urological Association already recommend PSMA PET/CT for patients after radical prostatectomy with increasing PSA level > 0.2 ng/mL, but only at a recommendation level of 2b. This review of the current state was intended to improve the evidence of clinical benefit and should contribute to strengthen the level of recommendation [52].

The overall ^18^F-PSMA-1007 PET/CT detection rate for local recurrence of this tracer was high in keeping with data reported by Hoffmann et al. (87.5%), Liu et al. (86%), and Watabe et al. (84.1%) [14,32,46]. Additionally, increasing detection rates with increasing PSA level was found, demonstrating high detection efficiency even in early recurrence, and compares favorably with other tracers [14,23,39].

For lymph node metastases, Sprute et al. reported accuracy data following histological follow-up of ^18^F-PSMA-1007 PET/CT lymph nodes in a mixed cohort of 96 patients with recurrent prostate cancer and primary prostate cancer, with a sensitivity and specificity of 81.7% and 99.6% and a PPV and NPV of 92.4% and 98.9%, respectively [13]. Similar results were seen in a study by Mingels et al. for pelvic lymph node metastasis ^18^F-PSMA-1007 PET/CT showed sensitivity 93%, specificity 92%, PPV 93% and NPV 92%, respectively [8].

Several studies reported a high rate of unspecified bone lesions in ^18^F-PSMA-1007 PET/CT. Dietlein et al. concluded that ^68^Ga-PSMA-11, ^18^F-DCFPyL or ^18^F-JK-PSMA-7 may be more suitable when there is suspicion on distant metastases, particularly in the bone marrow, because of their higher specificity in the bone marrow than ^18^F-PSMA-1007 [27].

One study assessed the change of management that can be obtained by using ^18^F-PSMA-1007 PET/CT. In a study by De Man et al. the patient-based detection rate was 82% and a management change was seen in 52% of the cases. The heterogeneous characteristics of the included patients resulted in a widely varying treatment change, mostly originating from an increase in disease extent on ^18^F-PSMA-1007 PET/CT [44].

The future perspective of recurrent prostate cancer diagnostics and therapy is also novel ligands. A recent study by Carlos Dos Santos et al. confirmed that radiolabeled ^64^Cu-PSMA is a promising agent to target and visualize PSMA receptor positive tumor lesions with high serum stability. This was shown in preclinical evaluation by small-animal PET studies, organ distribution and a patient application. Moreover, the images obtained at 20 h enabled delineation of unclear lesions, showing that the compounds fulfill the prerequisite for dosimetry in the course of future therapy planning with ^67^Cu ligand. It was suggested the ^64^Cu/^67^Cu-CA003 for clinical use for diagnostics and radiotherapy of prostate cancer, both primary and recurrent [53] as the theranostic pair. In order to support the ongoing research across Europe and beyond and to facilitate access to novel radionuclides, the PRISMAP consortium (European medical radionuclides programme www.prismap.eu (accessed on 28 October 2022).) was established in May 2021 to offer the broadest catalog of innovative radionuclides for medical research [54]. Development towards the upscaling of the production technology, new purification methods and proof-of-concept investigations may help to reach new diagnostic and treatment options for recurrent cancer patient care.

## 5. Conclusions

In conclusion, ^18^F-PSMA-1007 PET/CT appears to achieve reliable performance in detecting recurrent prostate cancer. The previous known high detection rate of ^18^F-PSMA-1007 PET/CT in recurrent prostate cancer was confirmed, especially in local recurrence and small lymph nodes with non-specific characteristics on conventional diagnostic imaging methods. However, it has some limitations, particularly for bone lesions, which appears a potential diagnostic limitation when using ^18^F-PSMA-1007 and the future perspective of recurrent prostate cancer diagnostics and therapy can also be reached by novel radionuclides and ligands.

## Figures and Tables

**Table 1:** Chemical structure of different PSMA ligands.

Ligand	Chemical Structure
^18^F-PSMA-1007	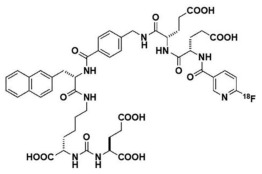
^68^Ga-PSMA-11	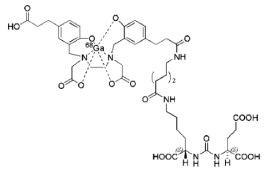
^18^F-JK-PSMA-7	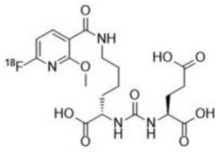
^18^F-DCFPyL	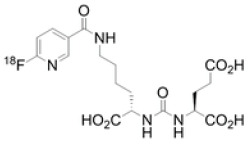

**Table 2:** Technical aspects of ^18^F-PSMA-1007 PET/CT.

Author	Year	Country	Mean Radiotracer Activity	Scanning Scope	Manufacturer of PET/CT	Mean Time Interval between Injection and Image Acquisition (min)
Mingels et al. [8]	2022	Switzerland	245.7 ± 13.3 MBq	From the head to the thighs	Biograph mCT PET/CT (Siemens, Erlangen, Germany)	120 ± 10
Morawitz et al. [24]	2021	Germany	229 ± 27 MBq	From the skull base to the mid-thigh	Biograph mCT 128 (Siemens Healthineers, Erlangen, Germany)	NM
Watabe et al. [14]	2021	Japan	259 ± 37 MBq	NM	Discovery 710 (GE, Milwaukee, WI, USA)	57.7 ± 4.9
Hoffmann et al. [32]	2022	Germany	NM	Whole body	Gemini TF16 PET/CT scanner (Philips Medical Systems, Best, The Netherlands)	90 ± 10
Giesel et al. [12]	2019	Germany, Chile	301 ± 6.46 MBq	NM	Biograph mCT Flow Scanner (Siemens Medical Solutions)	92 ± 26
Ahmadi Bidakhvid et al. [33]	2021	Belgium	3 MBq/kg	From the vertex to the upper thigh	Discovery MI-4 PET/CT (GE)	81 ± 16
Rauscher et al. [28]	2020	Germany	325 ± 40 MBq	NM	Biograph mCT scanner (Siemens Medical Solutions)	94 ± 22
Dietlein et al. [27]	2019	Germany	159 ± 31 MBq	NM	NM	NM
Rahbar et al. [34]	2018	Germany	338.02 ± 33.31 MBq	From the lower limbs to the skull	Siemens mCT Scanner (Siemens Healthcare, Knoxville, TN, USA)	Median 120
Sachpekidis et al. [35]	2019	Germany	Median 237 MBq	From the skull to the fee	Dedicated PET/CT system (Biograph mCT, 128S, Siemens, Erlangen, Germany)	60
Witkowska-Patena et al. [36]	2019	Poland	296 ± 14 MBq	From the top of the head to the mid-thigh	Dedicated hybrid PET/CT system (Discovery 710; GE Healthcare, Chicago, IL, USA)	95 ± 12
Wondergem et al. [37]	2021	The Netherlands	324 MBq	From the skull base to the inguinal region	Biograph-16 TruePoint PET/CT (Siemens Healthcare, Knoxville, TN, USA)	90
Saule et al. [29]	2021	Latvia	363.93 ± 69.40 MBq	From the head to middle thigh	Gemini TF64 (Philips, Koninklijke Philips N.V., Best, The Netherlands)	54

NM—not mentioned; MBq—megabecquerel; min–minutes.

**Table 3:** PET/CT performance in detection of local recurrence.

Author	Year	Country	Ligand	Patients (*n*)	Se	Sp	DR
Hoffmann et al. [32]	2022	Germany	^18^F-PSMA-1007, ^68^Ga-PSMA-11	128	-	-	87.5%
Liu et al. [43]	2022	Meta- analysis	^18^F-PSMA-1007	799	0.836	0.946	-
De Man et al. [44]	2022	Belgium	^18^F-PSMA-1007	51	-	-	82%
Ferrari et al. [38]	2021	Meta- analysis	^18^F-PSMA-1007	853	-	-	81.3%
Treglia et al. [23]	2019	Meta- analysis	^18^F-PSMA-1007	645			81%
Giesel et al. [12]	2019	Germany, Chile	^18^F-PSMA-1007	251	-	-	80.2%
Wang et al. [45]	2021	Meta- analysis	^18^F-PSMA, ^18^F-Choline, ^18^F-Fluciclovine	1706	-	-	83%
Liu et al. [46]	2022	Meta- analysis	^18^F-PSMA-1007	1002	-	-	86%
Mingels et al. [8]	2022	Switzerland	^18^F-PSMA-1007	177	0.95	0.89	-
Lengana et al. [42]	2021	South Africa	^18^F-PSMA-1007, ^68^Ga-PSMA-11	21	0.889	1	
Saule et al. [29]	2021	Latvia	^18^F-PSMA-1007	21	0.91	1	-
Watabe et al. [14]	2021	Japan	^18^F-PSMA-1007	28	-	-	84.1%

Se—sensitivity; Sp—specificity; DR—detection rate.

## Data Availability

Not applicable.
